# Effect of Changes to the Neighborhood Built Environment on Physical Activity in a Low-Income African American Neighborhood

**Published:** 2012-02-16

**Authors:** Jeanette Gustat, Janet Rice, Kathryn M. Parker, Adam B. Becker, Thomas A. Farley

**Affiliations:** Prevention Research Center at Tulane University School of Public Health and Tropical Medicine; Prevention Research Center at Tulane University School of Public Health and Tropical Medicine, New Orleans, Louisiana; Prevention Research Center at Tulane University School of Public Health and Tropical Medicine, New Orleans, Louisiana; Consortium to Lower Obesity in Chicago Children, Chicago, Illinois; New York City Department of Health and Mental Hygiene, New York, New York

## Abstract

**Introduction:**

Obesity is a public health problem that is due in part to low levels of physical activity. Physical activity levels are influenced by the built environment. We examined how changes in the built environment affected residents' physical activity levels in a low-income, primarily African American neighborhood in New Orleans.

**Methods:**

We built a 6-block walking path and installed a school playground in an intervention neighborhood. We measured physical activity levels in this neighborhood and in 2 matched comparison neighborhoods by self-report, using door-to-door surveys, and by direct observations of neighborhood residents outside before (2006) and after (2008) the interventions. We used Pearson χ^2^ tests of independence to assess bivariate associations and logistic regression models to assess the effect of the interventions.

**Results:**

Neighborhoods were comparable at baseline in demographic composition, choice of physical activity locations, and percentage of residents who participated in physical activity. Self-reported physical activity increased over time in most neighborhoods. The proportion of residents observed who were active increased significantly in the section of the intervention neighborhood with the path compared with comparison neighborhoods. Among residents who were observed engaging in physical activity, 41% were moderately to vigorously active in the section of the intervention neighborhood with the path compared with 24% and 38% in the comparison neighborhoods at the postintervention measurement (*P* < .001).

**Conclusion:**

Changes to the built environment may increase neighborhood physical activity in low-income, African American neighborhoods.

## Introduction

Obesity is a serious and widespread problem in the United States. Nearly 34% of American adults are obese, and 68% are either obese or overweight (1). In 2007, Louisiana had the third highest rate of obesity in the nation; according to self-reported data, 30.7% of adults were obese (2). Youth and adults in New Orleans are less likely to be physically active than those in the rest of the country (3). Among adults in New Orleans, 38% met the recommended levels of physical activity (PA) compared to 49% nationally. These findings were even more pronounced among low- and moderate-income African Americans (2).

Features of the built environment influence the propensity to be physically active (4-7). PA can be facilitated or constrained by the built environment, although the relationship between individual factors, social factors, and the physical environment is complex and not well understood (8). Making changes to the built environment should be considered as a means of addressing the related problems of obesity and physical inactivity (9). The Institute of Medicine has identified improvements to the built environment that can encourage walking and bicycling, such as a well-connected network of off-street trails and paths and paths connecting destinations for such activity, as a priority (10).

The Prevention Research Center (PRC) at Tulane University works to identify and address physical and social environmental factors that influence the obesity epidemic and has an overall goal of reducing obesity and its associated health problems. In this project, the Partnership for an Active Community Environment (PACE), the PRC worked with neighborhood-based community groups to create improvements to the built environment that would facilitate PA. Taking a community-based participatory research approach (11), we recruited members of local organizations to participate in the project steering committee. The objective of this study was to asses the effect of improvements to the built environment on the PA levels of residents in a low-income, African American community.

## Methods

We used a serial cross-sectional study design to evaluate the effect of the installation of a path and playground on community-wide PA. We conducted cross-sectional assessments at baseline (fall of 2006) and follow-up (fall of 2008). The changes to the built environment occurred in 2007. The study protocol received approval from the Tulane University institutional review board.

### Setting

In keeping with principles of community-based participatory research (11), we chose the PACE project intervention neighborhood on the basis of long-standing relationships between neighborhood leaders and Tulane University investigators (12). Meeting monthly, members of local community organizations and Tulane University faculty and staff formed the PACE steering committee (Appendix). The committee helped establish boundaries for the intervention neighborhood, identified 2 comparison neighborhoods, and chose to install a walking path and support the installation and use of a school playground as the built environment interventions. Neighborhoods were matched as closely as possible on proportion of homes owned by their residents, education level and annual household income of residents, percentage of African American residents, and similarity of the built environment, including housing and business type (13).

**Figure 1.**
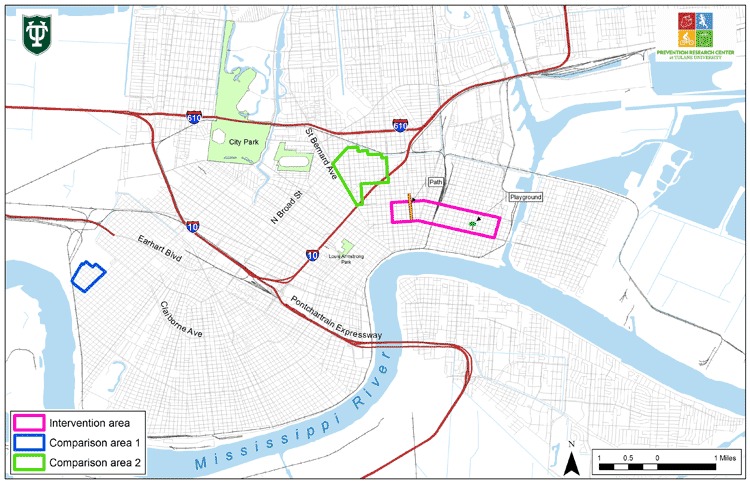
Partnership for an Active Community Environment Study Areas, New Orleans, Louisiana.

We also considered neighborhood flood levels at the time of Hurricane Katrina (August 29, 2005) to ensure that a similar level of damage and rebuilding in each of the neighborhoods existed (14). The chosen neighborhoods were reoccupied and in the process of being rebuilt by the time baseline assessments were conducted in the fall of 2006. All 3 neighborhoods were urban communities composed of single-family homes, apartments in old houses, small apartment complexes, and small businesses, and had sidewalks on nearly every street, although many were in poor condition. The intervention neighborhood and 1 comparison neighborhood each had a single large playground, both of which were taken over by the Federal Emergency Management Agency and used as a trailer park, making them unavailable for public use during the study period. There was no such space in the other comparison neighborhood. Streets and sidewalks were the only available places for PA in the neighborhoods. The schools in each neighborhood all had fenced, locked, concrete-slab yards with no playground equipment. One comparison neighborhood was approximately 1.5 miles and the other 5.4 miles from the intervention neighborhood. Because railroad tracks divided the intervention neighborhood, the steering committee recommended 2 interventions, 1 on each side of the tracks: the path in Area A and the playground in Area B (Figure 1).

### Intervention

The PACE steering committee chose to install a walking path in Area A of the intervention neighborhood (INA). In November 2007, PACE and the city of New Orleans built an 8-foot-wide path of 6 blocks on a grassy, tree-filled median of a wide neighborhood boulevard. The path connected a park outside the intervention area to a commercial corridor.

In another intervention, in May 2007, KaBoom! (http://kaboom.org), a national nonprofit organization, installed a playground with the help of community members and organizations on the back lot of a local elementary school in Area B of the intervention neighborhood (INB). The PACE project paid supervisors to keep the fenced playground open after school hours and on weekends from summer 2007 through spring 2009.

### Household survey

We measured self-reported PA in the intervention neighborhood and the 2 comparison neighborhoods through interviewer-administered household surveys conducted door to door (survey instruments available upon request to corresponding author). The sampling plan consisted of 2 separate stratified random samples of households before (September 2006 through February 2007) and after (October 2008 through January 2009) the walking path and the playground were built. A total of 6,497 households were in the 3 areas; 3,115 in the intervention neighborhood, 943 in the first comparison neighborhood, and 2,439 in the second comparison neighborhood. Trained interviewers orally administered the survey, which assessed the community social environment, the community physical environment, and self-reported PA, health and well-being, height, weight, and demographic characteristics. Interviewers randomly selected from each household 1 English-speaking adult aged 18 to 70 who had lived in the neighborhood for at least 3 months. Interviewers made up to 12 attempts to reach that person. People were excluded if they did not speak English, had not lived in the neighborhood for at least 3 months, or were outside the age range. PA questions covered walking for leisure, walking for transportation, and engaging in other activities such as bicycling or jogging. We also asked about use of specific locations for such activity.

At baseline, we sampled 778 households and conducted 499 interviews (response rate, 64.1%): 113 (out of 184) in INA, 111 (out of 174) in INB, 159 (out of 255) in comparison neighborhood 1 (CN1), and 116 (out of 165) in comparison neighborhood 2 (CN2). Of people sampled, we were unable to contact 112 (14.4%): 36 in INA, 21 in INB, 33 in CN1, and 22 in CN2. At follow-up, we sampled 900 households and conducted 692 interviews (response rate, 76.9%): 144 (out of 179) in INA, 192 (out of 253) in INB, 169 (out of 204) in CN1, and 187 (out of 264) in CN2. Of people sampled, we were unable to contact 109 (12.1%): 22 in INA, 26 in INB, 9 in CN1, and 52 in CN2.

### Physical activity observations

We used adapted SOPLAY (System for Observing Play and Leisure Activity in Youth) methods to objectively measure neighborhood PA on streets, sidewalks, and outside public areas on every block in each of the 3 neighborhoods (15). The basis of the SOPLAY system is momentary time sampling; it uses group time sampling techniques (15) and counts the number of people and their PA levels during play and leisure opportunities. Observers can use this technique in a neighborhood setting, where spontaneous activity of varying levels occurs among a changing number of people. Our interest was entire neighborhoods, so observers drove through neighborhood streets to count people. We established driving routes to cover all streets. Trained observers scanned the blocks, counting the numbers of sedentary, walking (moderate PA), and very active (vigorous PA) people, including youth and adult men and women. The protocol defined "vigorous" as any activity that was more active than walking, including running, lifting, bicycling, pushing, carrying, and dancing. We recorded and controlled for contextual factors such as weather. Unusual events such as street parties were noted. During observations, we divided the intervention neighborhood into 2 sections (INA and INB).

Data collectors conducted PA observations in the afternoons between 4:00 pm and 6:00 pm, 3 days per week (Thursday, Saturday, and Sunday), for 6 weeks (from October through early December, excluding the week of Thanksgiving) in 2006 and 2008.

### Analysis

We examined self-reported PA in several ways. Respondents indicated dichotomously (yes/no) whether they walked for transportation and walked for leisure (which included walking for exercise or walking a dog). We created 2 binary variables (1 for transportation and 1 for leisure) to indicate walking at least 30 minutes per day for at least 5 days per week and created a single binary variable to indicate walking for transportation, leisure, both, or not walking. Results were similar for the 3 approaches and were, therefore, only reported for the first. Frequencies of other forms of PA were low and were not included.

We compared neighborhoods at baseline and follow-up for the proportion of observed people that were moderately or vigorously active, for self-reported PA and location of activity, and for sociodemographic characteristics. We treated the 2 sections of the intervention neighborhood as separate neighborhoods, which required 3 dummy variables to code the intervention neighborhood.

We computed Pearson χ^2^ statistics to explore the bivariate relationships and used logistic regression to explore the effect of the intervention. We considered age as a confounder and potential effect modifier. Regression models included neighborhood, time, and neighborhood-by-time interactions. If the neighborhood-by-time interaction was significant, we used post hoc tests to determine whether the intervention neighborhood sections changed more than the comparison neighborhoods. We set significance at *P* < .05 and used SAS version 9.1 (SAS Institute, Inc, Cary, North Carolina) or Stata version 9.0 (StataCorp LP, College Station, Texas) for analyses. Everyone who took the survey completed it, and less than 5% of responses were missing. No imputation was conducted.

## Results

Survey respondents from the 3 neighborhoods were similar demographically at baseline (Table 1). Walking was the most common PA, and participation in any other activity was low (data not shown). Self-reported walking for both transportation and leisure increased from baseline to follow-up for most neighborhoods (Table 2). Because this increase was similar for each neighborhood, the neighborhood-by-time interactions were not significant. Age was neither a confounder nor an effect modifier (data not shown).

People from all neighborhoods reported that they most frequently exercised at sidewalks, mall or stores, and streets (Table 3). No significant neighborhood-by-time interactions were found. Walking trail use increased slightly but nonsignificantly (from 21.9% to 29.6%) in INA.

**Figure 2.**
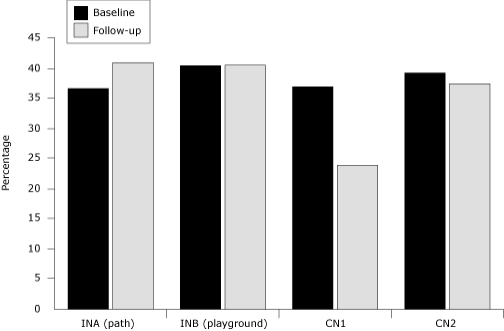
Percentage of people observed in intervention and comparison neighborhoods engaged in moderate and vigorous physical activity, at baseline (2006) and follow-up (2008), Partnership for an Active Community Environment Project, New Orleans, Louisiana. Neighborhood-by-time interaction was significant (χ^2^ test, *P* = .001), and differences between baseline and follow-up for INA and CN1 were significant (Pearson χ^2^ test, *P* < .001). Abbreviations: INA, Area A of intervention neighborhood (path); INB, Area B of intervention neighborhood (playground); CN1, comparison neighborhood 1; CN2, comparison neighborhood 2.

We found a significant neighborhood-by-time interaction between baseline and follow-up for the proportion of people observed who were active. A significant increase in the proportion of people engaged in moderate and vigorous activity was noted in INA between baseline (36.7%) and follow-up (41.0%) (Pearson χ^2^ test, *P* < .001) (Figure 2).

**Table:** 

Neighborhood	Baseline, %	Follow-Up, %
INA	36.7	41.0
INB	40.5	40.6
CN1	37.0	23.9
CN2	39.3	37.5

**Figure 3.**
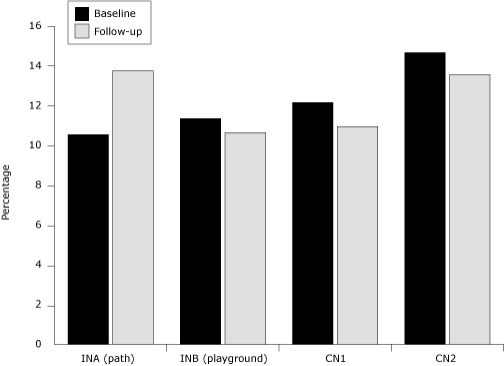
Percentage of people observed in intervention and comparison neighborhoods engaged in vigorous physical activity, at baseline (2006) and follow-up (2008), Partnership for an Active Community Environment Project, New Orleans, Louisiana. Neighborhood-by-time interaction was significant (χ^2^ test, *P* = .001), and the difference between baseline and follow-up for INA was significant (Pearson χ^2^ test, *P* < .001). Abbreviations: INA, Area A of intervention neighborhood (path); INB, Area B of intervention neighborhood (playground); CN1, comparison neighborhood 1; CN2, comparison neighborhood 2.

We observed a slight, significant increase in vigorous activity from 10.5% to 13.7% in that same area (Pearson χ^2^ test, *P* < .001) (Figure 3).

**Table:** 

Neighborhood	Baseline, %	Follow-Up, %
INA	10.5	13.7
INB	11.3	10.6
CN1	12.1	10.9
CN2	14.6	13.5

We analyzed the combined proportion of moderate and vigorous activity for youth and adults separately and the patterns of moderate and vigorous activity were similar (combined proportions shown) (Figure 2).

The playground was open for 81 weeks, from July 2007 through April 2009. The daily counts of playground users ranged from 1 to 114 (mean daily count, 25 users; data not shown).

## Discussion

We found that after a walking path was installed in a low-income neighborhood, the proportion of people observed who were active in that area increased. Observed activity decreased in the other areas during the same period. Self-reported activity also increased for residents in both intervention areas on streets, for everyone in parks, and for those in INA on a walking trail. However, only the change in park use was significant. The modest increases in observed activity around the path suggest that improvements to the built environment may have had this effect and are encouraging.

It must be noted that the evaluation of the path was not targeted around the path. We hypothesized that environmental changes available to the entire community would lead to increases in PA throughout the neighborhood, as norms for PA changed. However, we could not focus data collection where the built environment was altered because the interventions were decided after baseline data were gathered. Regardless, we were able to show a modest increase in both observed moderate and vigorous activity combined from 36.7% to 41.0% and in observed vigorous activity from 10.5% to 13.7%.

Randomized, controlled trials involving changes to the built environment are difficult to conduct, so most research has been cross-sectional. Our study used cross-sectional assessments before and after the interventions were implemented. In a review article relating features of the built environment to PA in majority African American populations, associations have not been consistent (16). We were unable to find other studies that used objectively measured PA to evaluate interventions to the built environment in primarily African American settings. Objectively measured activity is a better measure of the changes occurring in the neighborhood (17). We also saw some correspondence with the self-reported findings on increase in walking trail use. Our finding is encouraging and confirms cross-sectional studies that find a relationship between features in the built environment and an increase in PA.

Trails and other outdoor recreational spaces in the physical environment promote PA, as evidenced by this study and others (6,18,19). Community-based interventions such as implementing walking paths and trails are a strategy to increase PA levels of residents. A key component to a successful walking path or trail is the ability of residents to have access near their homes and to provide connectivity by linking to destinations (17,20-23). The path in this study was centrally located in the neighborhood and connected a well-used park to a commercial district with markets, shops, and businesses that the neighborhood residents frequent.

Environmental interventions aimed at increasing PA have shown modest results. Fitzhugh et al found an association between objectively measured PA and increased PA after installation of a greenway/trail (24). Their study placed observers in a stationary position in the neighborhood and only captured people being active at the observed location. Our methods allowed us to observe the activity of the entire neighborhood and to count sedentary as well as active people.

The study has several limitations. First, respondents tend to overestimate duration and intensity of PA when the accounts are self-recalled (25). However, systematic observations such as the ones we used are reliable measures of the use of a place or space (26) and may overcome the limitations of self-report (27). Second, when neighborhood is the unit of analysis, randomization is nearly impossible to accomplish, and matching for comparison is even more difficult than matching individuals, making it challenging to control for confounding variables. However, we chose 2 comparable neighborhoods, using census data and the subjective experience and perceptions of steering committee members. The data suggest that these efforts were successful and that communities were well-matched on key variables. Third, the PA observations were limited to streets and yards of houses. Parking lots, areas of commercial lots, and the fenced area of the school yard were not captured in the observations. We were not able to determine whether the users of the school playground were simply displaced from the streets where they may have otherwise played or were new users who were not previously playing outside. Fourth, evaluations of the neighborhoods were broad and encompassed the entire area both in sampling households throughout the neighborhood for the survey and in observing every street in each neighborhood for PA. A more targeted evaluation of the area where the interventions were implemented may have yielded a better measure of effect.

This study has several strengths. The study design included 2 comparison communities, subjective and objective measures of the outcome variables, and 2 waves of data collection. The communities were chosen using objective considerations as well as via the participation of members of the steering committee, who had "insider" perspectives. The interventions were community generated or supported, making them more likely to be accepted and used (11). Contextual variables unique to the time and place (post-Katrina New Orleans) were carefully considered and effectively measured.

Built environment changes, such as easily accessible paths that lead to destinations, can provide more opportunities for PA in primarily African American neighborhoods and others where infrastructure has been allowed to fall into disrepair or was not initially installed. Such increases in opportunity can lead to increases in population-level PA. Environmental interventions can affect many people, over a long period, which programmatic interventions cannot do. Cities and planning commissions should prioritize places for people to be active that are in or near the neighborhoods where people live and consider simple adaptations to existing infrastructure. Resident participation in decision making about the nature and location of environmental changes may be necessary to ensure that changes lead to changes in behavior. As PA levels decrease across the country and in specific communities, creating supportive environments for activity is important. Walking paths or trails may provide a long-term, sustainable, and low-cost strategy to increase the PA of residents in low-income neighborhoods.

## Figures and Tables

**Table 1.** Baseline Demographic Data and Self-Reported Body Mass Index (BMI), Household Survey Respondents in Intervention and Comparison Neighborhoods, New Orleans, Louisiana, Partnership for an Active Community Environment Project, 2006

**Characteristic**	INA (n = 105)^a^	INB (n = 108)	CN1 (n = 150)	CN2 (n = 110)
**African American, %^b^**	85.7	91.7	96.7	100.0
**Female, %**	54.7	63.9	65.3	60.4
**Employed, %**	61.2	50.9	49.7	60.6
**≥GED/high school graduate, %**	82.9	76.2	80.4	88.3
**Age, mean (SD),^c^ y**	41.6 (14.3)	47.0 (14.0)	43.5 (13.6)	45.5 (14.1)
**Annual income ≥$20,000, %**	36.0	46.7	33.6	46.2
**BMI, mean (SD), kg/m^2^**				
Male	27.9 (6.7)	27.6 (6.8)	26.3 (4.8)	26.8 (5.0)
Female	27.7 (8.0)	29.6 (6.0)	30.0 (8.5)	30.1 (7.6)

Abbreviations: INA, intervention neighborhood A (path); INB, intervention neighborhood B (playground); CN1, comparison neighborhood 1; CN2, comparison neighborhood 2; GED, general educational development certificate; SD, standard deviation.

a Differences between total numbers in table and methods section result from item nonresponse.

b Difference between neighborhoods at baseline, *P* < .001 (Pearson χ^2^ test).

c Difference between neighborhoods at baseline, *P* = .03 (Pearson χ^2^ test).

**Table 2.** Self-Reported Walking at Baseline (2006, n = 476) and Follow-Up (2008, n = 665),^a^ by Neighborhood, New Orleans, Louisiana, Partnership for an Active Community Environment Project^b^

Neighborhood	Walk for Transportation, %		Walk for Leisure, %	

	Baseline	Follow-Up	Baseline	Follow-Up
Intervention A (path)	29.3	34.8	60.0	65.3
Intervention B (playground)	24.8	36.9	63.3	61.5
Comparison 1	31.3	40.5	61.3	70.4
Comparison 2	19.8	31.1	57.7	68.9

a Differences between total numbers in table and methods section result from item nonresponse.

b Neighborhood-by-time interaction was not significant.

**Table 3.** Percentage of Survey Respondents Who Reported Exercising at Specific Locations, by Neighborhood, at Baseline (2006, n = 473) and Follow-Up (2008, n = 666), New Orleans, Louisiana, Partnership for an Active Community Environment Project^a^^,^ b

Location	Baseline, %				Follow-Up, %			

	INA	INB	CN1	CN2	INA	INB	CN1	CN2
Sidewalk	61.5	53.2	60.0	51.8	52.1	52.9	54.4	52.8
Mall or store^c^	35.2	45.0	54.7	47.3	31.9	38.7	34.4	40.6
Street	40.4	32.1	46.7	42.7	41.5	41.7	41.9	37.2
Park^c^	27.6	14.7	30.7	18.2	34.5	26.2	38.4	36.5
Walking trail	21.9	21.1	25.3	17.3	29.6	15.0	21.4	20.8
Equipment in home^c^	21.9	23.8	19.3	22.7	13.4	13.4	11.9	14.4
Indoor gym^d^	21.9	18.4	21.5	18.2	11.4	9.6	13.1	18.3
Track at local school	10.5	8.3	9.3	9.1	6.3	4.3	6.3	14.4

Abbreviations: INA, intervention neighborhood A (path); INB, intervention neighborhood B (playground); CN1, comparison neighborhood 1; CN2, comparison neighborhood 2.

a Differences between total numbers in table and methods section result from item nonresponse.

b There were no significant neighborhood-by-time interactions.

c Overall differences between baseline and follow-up, *P* < .001 (Pearson χ^2^ test).

d Overall differences between baseline and follow-up, *P* = .002 (Pearson χ^2^ test).

## Appendix

### Appendix. Member Organizations Represented in the Partnership for an Active Community Environment Steering Committee

1. All Citizens Together
2. Bunny Friend Neighborhood Association
3. Bywater Hospital (2004-2005)
4. Crescent City Peace Alliance
5. Dillard University (2004-2005)
6. Douglass Community Coalition (2004-2005)
7. Faubourg St. Roch Improvement Association
8. Holy Angels/Marionites
9. Small Axe Urban Farms
10. St. Claude Merchants' Association (2004-2006)
11. St. Roch Neighborhood Association
12. St. Roch Community Church
13. Steps to a Healthier Louisiana (a project of the New Orleans City Health Department and the Louisiana Public Health Institute)
14. The Renaissance Project (2004-2007)
15. Tulane University School of Public Health and Tropical Medicine
16. Urbanheart (2004-2005)
